# Perceptions of factors influencing Ebola vaccine acceptance among community members, healthcare workers, and response personnel in Eastern Democratic Republic of the Congo

**DOI:** 10.1371/journal.pone.0346572

**Published:** 2026-04-07

**Authors:** Ruth Kallay, Pierre Muhoza, Dieula Delissaint Tchoualeu, Monica Fleming, Stephanie Garbern, Samuel Lwamushi Makali, Shiromi M. Perera, Rigo Fraterne‐Muhayangabo, Arsene Baleke Ombeni, Alexandra Tuttle, Emma Aberle-Grasse, Nancy Ortiz, Reena Doshi, Adam C. Levine, Eta Ngole Mbong

**Affiliations:** 1 Global Immunization Division, U.S. Centers for Disease Control and Prevention, Atlanta, Georgia, United States of America; 2 Warren Alpert Medical School of Brown University, Providence, Rhode Island, United States of America; 3 Regional School of Public Health, Catholic University of Bukavu, Bukavu, Democratic Republic of Congo; 4 International Medical Corps, Washington, DC, United States of America; 5 International Medical Corps, Goma, Democratic Republic of the Congo; Kampala International University - Western Campus, UGANDA

## Abstract

**Background:**

The 2018–2020 Ebola Virus Disease (EVD) outbreak in Eastern Democratic Republic of the Congo (DRC) occurred amid armed conflict, institutional mistrust, and fragile health systems. The Ebola vaccine was deployed under emergency pre-licensure use, and concerns about it persisted. This study explored community and healthcare worker (HCW) perceptions of the Ebola vaccine to better understand the sociocultural and structural drivers of vaccine acceptance.

**Methods:**

We conducted a qualitative study in three heavily affected health zones in North Kivu province (Beni, Butembo, and Mabalako) in 2021. Data were collected through thirty-three focus group discussions and 15 key informant interviews with EVD survivors, community members, HCWs, and local leaders, purposively sampled to capture diverse perspectives. Transcripts were analyzed using thematic and content analysis.

**Results:**

Participants reported concerns about the safety of the vaccine, mistrust in the institutions delivering it, and confusion due to rumors and inconsistent communication from the Ebola response. HCWs reported feeling coerced into vaccination rather than making a voluntary choice. Misinformation, logistical barriers, and perceptions of favoritism and stigmatization linked to ring vaccination were cited as preventing acceptance. Religion played a dual role, both fostering skepticism and encouraging acceptance depending on the stance of local faith leaders. Participants emphasized the need for transparent and balanced communication, equitable access, and greater involvement of trusted and competent community figures in vaccination efforts.

**Conclusions:**

Ebola vaccine decision-making in Eastern DRC was shaped by complex interactions between institutional mistrust, perceived risk, religion, and access constraints within a broader context of sociopolitical instability. This study provides a critical baseline of perceptions during the vaccine’s pre-licensure phase and highlights the importance of locally grounded engagement strategies. As vaccines become licensed, understanding local perceptions as well as leveraging the influence of trusted religious and community leaders will be essential for improving vaccine uptake.

## Introduction

Since the identification of the Ebola virus in the Democratic Republic of the Congo (DRC) in 1976, the country has experienced recurrent Ebola Virus Disease (EVD) outbreaks, with increasing frequency in recent years [1]. The 2018-2020 EVD outbreak in Eastern DRC was the largest in the country’s history and the second largest EVD outbreak globally, resulting in over 3,481 cases (3,323 confirmed, 158 probable) and a case fatality rate of 66% [2]. At the time, only experimental Ebola vaccine doses were available for use in the DRC, administered under an Expanded Access/Compassionate use protocol as recommended by the World Health Organization (WHO)’s Strategic Advisory Group of Experts on Immunization (SAGE) [3,4]. The vaccine was deployed using a ring vaccination strategy, which involved vaccinating contacts and contacts of contacts of EVD cases to interrupt disease transmission [5]. However, the scale and geographic extent of the outbreak exposed critical challenges in the Ebola response and revealed diverse community reactions to vaccination efforts.

The response was also challenged by the complex realities of Eastern DRC, including active conflict, population displacement, a fragile health system, and widespread distrust of both government and foreign actors [6–8]. Although the ring vaccination strategy was implemented, uptake was hindered by persistent community resistance and fears about the vaccine’s safety and intent. Misinformation and rumors, such as suspicions that the vaccine might cause Ebola, led to refusals and, in some cases, violence against response personnel [7]. While trust in institutions was identified as a predictor of vaccine acceptance during the early phase of the outbreak, the sociopolitical context, including historical marginalization and unmet basic needs, continued to shape public skepticism [7]. Community feedback mechanisms provided broad insights into public sentiment, but there remains a lack of in-depth understanding of the local drivers of Ebola vaccine acceptance, especially among healthcare workers and affected communities in Eastern DRC.

Insights from the 2014–2016 West Africa Ebola outbreak highlighted the complex behavioral drivers behind vaccine acceptance, including altruistic motivations, perceived risk, trust in response teams, and broader concerns related to power, fairness, and social justice [8]. Given the increasing number of outbreaks and new focus on preventive Ebola vaccine use, there is a need for updated insights into community perceptions of the vaccine [1,9,10]. Understanding these perceptions is vital for informing effective strategies to improve vaccine uptake in both current and future outbreak responses.

The WHO SAGE vaccine hesitancy working group defines vaccine hesitancy as the delay in acceptance or refusal of vaccines despite the availability of vaccination services [11]. To better understand the roots of hesitancy, the SAGE working group developed the confidence, complacency, and convenience (i.e., “three Cs”) model which posits that vaccine hesitancy arises from (i) a lack of *confidence* in the safety and effectiveness of the vaccine and the system recommending and delivering it; (ii) *complacency*, or the belief that the disease poses minimal risk and vaccination is unnecessary; and (iii) *convenience* barriers, such as difficulties with access, affordability, or the appeal of vaccination services [12]. Vaccine hesitancy is shaped by a complex interaction of cultural, social, psychological, and systemic factors, particularly in the context of EVD outbreaks that strain healthcare infrastructure and challenge public trust.

In the DRC, where EVD outbreaks remain a recurrent threat, it is critical to understand the local sociocultural and contextual factors that shape vaccine perceptions. Comprehensive qualitative research can provide valuable insights into these dynamics, helping bridge the gap between public health recommendations and community realities. This study aimed to explore the factors influencing Ebola vaccine acceptance among communities affected by the 2018–2020 EVD outbreak in eastern DRC. Additionally, we sought input from both community members and key informants on strategies to strengthen vaccine delivery and improve future outbreak response efforts. The findings are intended to inform the design of community-centered approaches that build trust, promote engagement, and address vaccine hesitancy in meaningful and locally relevant ways.

## Methods

### Study design

This study focuses on the qualitative component of a broader mixed methods study aiming to understand community perspectives on Ebola vaccine hesitancy and acceptance in North Kivu province, DRC [13]. The qualitative study was conducted as a follow-up to elaborate and explain the quantitative study which has been described elsewhere [14,15]. The qualitative component included focus group discussions (FGDs) and key informant interviews (KIIs).

### Study setting and study participants

This study was conducted in three health zones of North Kivu province (Beni, Butembo, and Mabalako) selected for their high EVD transmission during the 2018–2020 outbreak, the country’s 10^th^ per the ministry of health (MoH). North Kivu also shares porous borders with Uganda and Rwanda and has experienced three decades of conflict-related atrocities perpetrated by both foreign and local armed groups [16,17].

Study participants were purposively selected from each of the three health zones. They included EVD survivors and community members residing within the same area as the selected survivors during the 10^th^ EVD outbreak. Community members were recruited based on geographic proximity to survivor households, reflecting the social and neighborhood networks targeted during the ring vaccination strategy. Survivors were the entry point for the preceding quantitative phase to help identify individuals likely offered the Ebola vaccine as known case contacts. Ebola virus disease survivors were identified through a local voluntary survivor support association that maintains a registry of individuals affected during the outbreak; survivors included in the broader mixed-methods project were randomly selected from this registry during the quantitative phase. Additional details on the survivor identification and sampling procedures used in the quantitative phase of the mixed-methods study have been published elsewhere [18]. Each FGD had a different composition of participants across community roles (i.e., general community members, community leaders, religious leaders, traditional healers, HCWs and other response personnel), sex, Ebola vaccination status, and health zone. Vaccination status referred to receipt of the rVSV-ZEBOV (Ervebo) Ebola vaccine used during the 10th EVD outbreak in these zones. FGDs for vaccinated and unvaccinated participants and for men and women were held separately within each community role, to encourage open and transparent discussion. Mixed vaccination status groups were formed only when there were insufficient numbers in either group to do separate FGDs. KIIs were conducted with individuals holding a leadership role in the community during the assessed Ebola outbreak or in governmental or nongovernmental organizations involved in Ebola vaccination during that period.

### Data collection

Data collection activities included FGDs and KIIs, conducted during July 27 and August 10 2021. These activities were conducted in-person and in the participants’ language of choice by data collectors trained in qualitative research and fluent in French, Swahili and Kinande, the predominant local languages. This occurred during ongoing COVID-19 transmission and thus safety procedures included social distancing and the use of face masks. Interviewers obtained verbal informed consent from all participants prior to data collection.

Inclusion criteria were age over 18 years, residency in one of the targeted health zones (Beni, Butembo, or Mabalako) during the (DRC’s 10th Ebola outbreak, and willingness to provide verbal informed consent. Participants in FGDs were either survivors of the 10th Ebola outbreak or neighbors of survivors (likely case contacts) who were part of the community group prioritized for vaccination with the limited doses of the rVSV-ZEBOV Ebola vaccine available at the time. HCWs and frontline workers (FLWs) were included only if they had worked in a health facility within the targeted health zones during the outbreak and were among those prioritized for vaccination. In this study, ‘frontline workers’ refers to individuals identified during recruitment as being directly involved in the Ebola response, but not necessarily in clinical roles. Key informants were individuals who held leadership roles during the outbreak, either in the community or within governmental or nongovernmental organizations involved in the Ebola vaccination effort. All interviews were audio-recorded with permission and field notes were taken. FGDs lasted approximately 90 minutes, and KIIs about 60 minutes.

We used a semi-structured guide for the FGDs and KIIs to enable the exploration of a consistent set of questions while at the same time providing the flexibility to probe topics specific to the respondents’ community role and Ebola vaccination status. Participants were asked questions around topic areas expected to influence vaccine uptake including perceived risk of disease; communications (including rumors, misinformation, sources of information, community engagement); societal factors (including religion, community leaders, government, security concerns); health systems (e.g., vaccination teams, geography, logistics); and vaccine specific factors (e.g., safety and efficacy). These topic areas were informed by a literature review, community feedback documented in reports by local health authorities during the outbreak, and direct input from those authorities. Depending on their Ebola vaccination status, respondents were asked their reasons for accepting or refusing vaccination when offered.

The participant characteristics, by health zone, for the 33 FGDs and 15 KIIs that were conducted across Beni, Butembo, and Mabalako health zones are shown in Tables 1 and 2, respectively. A total of 263 participants participated in the FGDs, with some variation by health zone.

**Table 1:** Focus Groups conducted by health zone, Democratic Republic of the Congo, 2021. *Values represent number of FGDs; numbers in parentheses indicate number of participants.*

Category	Beni	Butembo	Mabalako	Total
Vaccination status				
Vaccinated	4 (33)	4 (30)	4 (33)	**12 (96)**
Unvaccinated	3 (25)	3 (21)	1 (16)	**7 (62)**
Mixed vaccination status	6 (44)	5 (39)	3 (22)	**14 (105)**
Sex				
Men	6 (46)	5 (39)	5 (38)	**16 (123)**
Women	7 (56)	7 (51)	3 (33)	**17 (140)**
Community roles				
Community members	4 (34)	4 (31)	3 (24)	**11 (89)**
Healthcare workers	3 (25)	3 (20)	2 (17)	**8 (62)**
Community leaders	2 (14)	2 (18)	2 (22)	**6 (54)**
Religious leaders	2 (12)	1 (7)	1 (8)	**4 (27)**
Traditional healers	2 (17)	2 (14)	0 (0)	**4 (31)**
Total focus groups conducted	**13 (102)**	**12 (90)**	**8 (71)**	**33 (263)**

**Table 2:** Key Informant Interviews conducted by health zone, Democratic Republic of the Congo, 2021.

Key Informant Category	Beni	Butembo	Mabalako	Total
Local government representatives¹	1	1	2	4
Community leaders²	0	0	1	1
Health system officials³	1	2	0	3
Ebola vaccination leads⁴	1	2	0	3
Humanitarian/NGO staff⁵	1	2	0	3
**Total Key Informant Interviews**	**4**	**7**	**4**	**15**

^1^Local government representatives include mayors, village chiefs, and police officials.

^2^Community leaders include presidents of community groups or similar local representatives.

^3^Health system officials include health zone chief doctors, health zone surveillance focal points, and routine immunization program lead nurses.

^4^Ebola vaccination leads include vaccination team leads and Ebola vaccination coordinators.

^5^Humanitarian/NGO staff include personnel affiliated with non-governmental organizations, such as WHO, Médecins Sans Frontières, and other partners.

### Data management and analysis

FGDs and KIIs were audio recorded, transcribed, and translated into English. The transcriptions and the field notes were organized, reviewed and analyzed using an open-source qualitative software, *Taguette*. To further manage the data and explore the prevalence of different themes and codes quantitatively, Microsoft Excel was also used.

We used a deductive approach for data analysis with codes that were guided by the interview guide and the vaccine hesitancy determinants matrix described by the three Cs model [12]. We selected this model for its established use in outbreak settings and its clear structure for identifying key behavioral drivers relevant to vaccine deployment [19–21]. Thematic analysis was conducted to distill overarching themes that emerged from the data. Each transcript was coded by 2 researchers, with discrepancies in interpretation resolved by a third reviewer. An iterative approach with multiple reviewers was used to ensure consistency and analytical rigor. Key themes reflecting the range of participant perspectives were identified collaboratively and aligned with the three Cs framework.

### Ethical considerations

The protocol was approved by the Ethics Committee of the Kinshasa School of Public Health (protocol approval #203−2020). Given that the assessment was conducted during the COVID-19 pandemic, the Ethics Committee also waived the requirement of written informed consent for participation. Verbal informed consent was obtained from all participants and documented electronically, due to low literacy rates and the need to limit physical contact during the COVID-19 pandemic. Participation was anonymous, voluntary, uncompensated, and had no impact on access to health services or survivor support. Prior to data collection, authorization was obtained from the Provincial Health Division of North Kivu and the respective Health Zone authorities (Médecins Chefs de Zone) in Beni, Butembo, and Mabalako. No individual community representative provided consent on behalf of participants; all participants provided their own informed consent as described above. The U.S. Centers for Disease Control and Prevention Global Health Center Human Subjects Office reviewed the protocol and deemed this a non-research public health activity [45 CFR 46.102(l)(2)]. This activity was conducted consistent with applicable federal law and CDC policy (45 C.F.R. part 46, 21 C.F.R. part 56; 42 U.S.C. Sect. 241(d); 5 U.S.C. Sect. 552a; 44 U.S.C. Sect. 3501 et seq.).

## Results

Thematic analysis yielded several predominant topic areas across FGDs and KIIs including barriers to vaccination, misinformation, sources of information, vaccine confidence, perceptions of the response teams, and reasons for vaccination, and community-based recommendations for improving vaccine confidence. These topic areas were then interpreted and presented using the “three Cs” model of vaccine hesitancy framework, which organizes the findings under the constructs of Vaccine Confidence, Complacency, and Convenience.

### Confidence

Aspects related to “confidence” emerged as the most salient drivers of Ebola vaccine acceptance. Respondents often mentioned issues related to trust in the safety and effectiveness of the Ebola vaccine, trust in the vaccination strategy used to deliver the vaccine, and trust in the health authorities and workers who develop and deliver vaccines. The main emergent themes are listed below.

#### Reasons for vaccination including risk perception, social influence, and professional obligation.

KII participants generally expressed a desire to serve as models for others by accepting the vaccine. They believed community members were likely to get vaccinated after seeing others do so without complications. They viewed the vaccine as a “*free*” or “*valuable”* opportunity to protect themselves, believing it reduced the severity of the disease. Many of these participants were exposed to Ebola through their work as senior HCWs leading their peers or in leadership positions within the community.

FGD participants generally reported being vaccinated because they viewed the vaccine as a protective measure and trusted the information provided by HCWs. Many also expressed feeling compelled to vaccinate due to employment-related requirements. This sentiment was especially prevalent among HCWs, some of whom stated that they risked losing their jobs if they chose not to get vaccinated. One participant noted:

*“If we refused taking the vaccine against Ebola, we ran the risk of losing our jobs because it was a principal condition to work as a nurse during the period of this epidemic.*”

- Male Healthcare Worker, Beni FGD

Similar requirements were reported by participants in other public-facing professions, such as the police and military, who also indicated that vaccination was mandatory for their roles during the outbreak.

In contrast, community members who were not in such roles were offered the vaccine based on their identification as likely contacts in line with the ring vaccination strategy. While not mandatory, some of these individuals still perceived the offer as coercive, particularly when presented with urgency or pressure from authorities. One participant shared:

*“They forced me to take the vaccine with threats against me and my whole family and my whole church because there was a confirmed case detected in my church.”*

- Male Religious Leader, Beni FGD

#### Vaccine rumors and misinformation.

Rumors and misinformation about the Ebola vaccine emerged as significant barriers to vaccination. Commonly cited barriers from FGDs and KIIs included fear of side effects, fear due to rumors, religious beliefs contributing to Ebola vaccine hesitancy, lack of confidence in the vaccine, safety concerns around vaccinating those with comorbidities or during pregnancy, and some HCWs discouraging vaccination against EVD.

Reports of deaths among vaccinated individuals (though participants could not provide evidence linking these deaths to the vaccine) fueled persistent rumors, including claims that the vaccine could cause death five years after immunization. Other common misinformation included the beliefs that the vaccine could lead to reproductive health issues such as infertility or impotence, and increased occurrence of miscarriages among vaccinated women. Fear of vaccine side effects and rumors were also attributed to: 1) poor community sensitization, including lack of adequate outreach and unclear or insufficient messaging about the vaccine, 2) negative feedback received from peers who had been vaccinated, and 3) fear of being forced into isolation in an Ebola treatment unit (ETU) if they exhibited Ebola-like symptoms which participants believed the vaccine could cause. These fears were compounded by inadequate communication about adverse events that could occur following immunization and a general misunderstanding of what occurred in the ETUs. There was a widespread fear of isolation in the ETU, fueled by the perception that most individuals who were taken died or were unable to communicate with families and friends during treatment. For example, one participant stated that:

*“The others were saying that this is the vaccine in which there is Ebola because some people who took this vaccine would get sick and when we brought them in to the [Ebola Treatment Unit], they died directly.”*

- Male Healthcare Worker, Beni FGD

Another participant noted:

“*I was worried that I would develop signs of Ebola and must be brought to the [Ebola Treatment Unit].”*

- Vaccinated male Healthcare Worker, Beni FGD

And yet another:

“*During this time, anyone who went to the hospital did not come home alive.”*

-Female Traditional Healer, Beni FGD

Barriers to vaccination varied across sex and vaccination status subgroups. Among men, general fear of side effects and concerns about the vaccine’s experimental nature were the most prominent issues. In contrast, women were more likely to express fears about vaccine-related mortality.

“*Ebola deaths occurred among those vaccinated. We said to ourselves that if a vaccinated person is dying, is it worth it to be vaccinated?*”

- Vaccinated female HCW, Mabalako FGD,

*“I did not trust this information because we were told the vaccine is experimental and that we had to sign a consent form before receiving it. To us, it felt like we were being treated as guinea pigs for vaccine experimentation.”*

- Non-vaccinated male HCW, Beni FGD

#### Questions and concerns about evolving vaccination strategy and processes.

The deployment of the Ebola vaccine through ring vaccination strategy negatively affected people’s perception of the vaccine. Participants noted that differences between the Ebola vaccination approach and routine immunization practices fueled misinformation and distrust. These differences included the use of incentives for identified contacts, the requirement of written consent, and the involvement of many frontline staff perceived as “foreign” (including non-local Congolese and international staff) in the Ebola response and vaccination. FGD participants often mentioned these as reasons for increased skepticism and doubts about the motives behind Ebola vaccination.

“*Have you seen a person after taking [a] vaccine receive the bag of rice and cooking oil. For me, they wanted to take advantage of our poverty and our ends to kill us with this vaccine.”*

- Non-vaccinated male, Beni FGD

Moreover, the ring vaccination strategy was perceived to create social stigma, as individuals associated with an EVD case (either directly or indirectly) were singled out for vaccination.

“*When you [were identified as] a contact case, there was too much mobilization. Your name would be posted at the entry points as if you were a rebel.*”

- Non-vaccinated male, Beni FGD

Because those already exposed to EVD were prioritized, some community members attributed subsequent illness or death among them to the vaccine itself. Unlike routine vaccines, which are typically administered preventively, participants questioned why the Ebola vaccine was offered only after exposure to a confirmed case.

“*Vaccination was a failure because they would vaccinate a family after the case had been declared positive, yet they should have vaccinated before the case was declared.”*

- Male Traditional Healer, Beni FGD

Changes to vaccine eligibility criteria over time, particularly the inclusion of previously ineligible populations, were met with criticism from the community. The revision of guidelines during the ongoing outbreak, to include groups previously considered ineligible, such as pregnant women, lactating women, and children over 6 months, led some participants to question the extent of health experts’ knowledge about the vaccine. Even when guideline updates were communicated, communities perceived inconsistent recommendations, which further fueled concerns about the vaccine’s safety and efficacy.

*“Sometimes they said that pregnant and breastfeeding women cannot have the vaccine, other times they said they can have it. So, I was afraid because of all the contradictions."*

- Non-vaccinated female, Beni FGD

#### Distrust in vaccine due to perceived politicization of Ebola response.

The politicization of the EVD response emerged as a recurring theme underlying many of the rumors circulating within the community. These rumors often suggested sinister motives behind the Ebola outbreak response and the vaccination campaign. Many participants viewed the Ebola vaccine as a tool intended to harm certain groups of people. Skeptical views ranged from the belief that some local ethnic groups were being targeted with the vaccine for extermination, to the idea that it was a tool used by foreigners to exterminate Africans. Participants also linked their distrust of the vaccine to skepticism toward authorities who prioritized vaccination over other urgent issues like civil unrest driven by poor governance and violence from armed groups believed to be from neighboring countries. This deepened suspicion toward the motives of frontline workers, government institutions, and other organizations involved in implementing the Ebola vaccination campaign. As one participant noted:

*“95% of people have negative feelings towards the vaccine because we are in a context where the government is ineffective and there is widespread suffering among the population. I heard the [rebels] considered vaccinated people as already ‘dead’, and some members of the government were complicit and did not want to give us accurate information about this vaccine.”*

- Non-vaccinated male, Butembo FGD

Another participant asserted:

*“Some politicians said that it was [a senior political figure] who had brought Ebola to the region to kill people. The outbreak appeared at a moment when people were already worn down by the massacres.”*

- Community Leader, Beni KII

There was a perceived bias in the handling of the Ebola response, particularly regarding the allocation of resources. Some participants compared the extensive resources mobilized for Ebola to the minimal attention given to other local health concerns or epidemics. Criticism was also directed at the “*flashy*” displays of wealth by response teams, especially by “*outsiders*”, but also locals who were perceived to have joined the response for financial gain. The Ebola response and the vaccination activities were frequently referred to as a “*business*”, “*manufactured crisis*”, or money-making scheme designed to benefit outsiders at the expense of local communities. For example, one participant asserted:

*“We couldn’t trust the vaccine because we saw people dying daily and a lot of people were coming here in cars. We could count more than 100 cars. And when you see such convoys, doubts start to sink in. We would wonder how come they hadn’t mobilized that much money when the disease was already here. This led us to believe there was some kind of business surrounding the vaccine*.”

- Community Leader, Beni KII

#### Trusted sources of information influencing attitudes towards the Ebola vaccine.

Overall, radio was the most frequently cited source of information among FGD participants. Community members and traditional healers also often identified churches as an additional key source of information, while HCWs tended to rely on guidance from WHO, the MOH or their HCW peers. Community and religious leaders most frequently mentioned community members, neighbors and sensitization efforts as their key sources of information.

*My sources of information on Ebola and its vaccination were the church and the radio. And personally, I trusted the information from my church more.*

- Female Traditional Healer, Butembo FGD

Radio and community sensitization activities were the primary sources of information for vaccinated FGD participants compared to church as the predominant source of information among non-vaccinated participants.

#### Recommendations for improving community awareness, trust and involvement.

Overall, participants agreed that improving community awareness through enhanced sensitization efforts would increase trust in the safety and effectiveness of the Ebola vaccine. Frequently mentioned recommendations included the need for reassurances to be clearly communicated by national health authorities that the vaccine has been tested and approved by them, and for transparent communication explaining not just the benefits of the vaccine but also its potential side effects, and their management. It was also commonly recommended that communications should be conducted in local languages, emphasize that vaccination is a voluntary choice, and that information about the vaccine should come from credible national authorities such as health experts and national health laboratories and not just community health volunteers.

*“Our health authorities had told us that this vaccine was experimental…*. *Knowing that this vaccine has been certified by the health authorities in our country encouraged vaccination.”*

- Male Healthcare Worker, Beni FGD

Another frequent recommendation was to increase local community involvement in the response and vaccination efforts. Suggestions included using trusted community figures – such as religious leaders, local village chiefs, heads of community groups, youth groups, and *cellules d’action communautaires* (Community Action Committees) – to lead sensitization efforts. These figures were seen as crucial for facilitating information sharing and ensuring that the concerns and needs of the community were addressed during the process.

*“I encourage you in this approach. The community must be consulted and involved before any intervention in its favor be it new vaccines, new strategies, etc.*"

- Vaccinated male Healthcare Worker, Butembo FGD

Respondents, however, stressed that locally recruited vaccinators and response workers should be properly qualified for their roles. While many expressed greater trust in local actors to provide information and vaccination services, they also emphasized that only those with relevant healthcare experience or proper training should be the ones to serve as communicators or to support vaccination activities, to ensure the credibility of the response. Participants noted that when local informal sector workers were included in the response, despite lacking relevant expertise and without support from local health experts, it eroded trust in the validity of the services and information provided by the vaccination teams.

“*In the response there were people…that we know very well were not in the [health] field, people such as masons and hairdressers who were in the response only because they knew highly placed personnel coordinating the response. This led us to doubt all response staff and even the existence of the epidemic.*"

- Non-vaccinated male, Butembo FGD

### Complacency

Community perceptions of Ebola and the vaccine shaped by beliefs about risk, religion, and trust in the response were central to vaccine acceptance and hesitancy.

#### Perceptions about Ebola vaccine and response.

Beliefs about the existence of Ebola varied among participant groups. While most participants acknowledged that EVD was real, others expressed skepticism or felt that they were not at risk. Among the non-vaccinated group, there was a general sense of indifference, with some stating things like “*there is no rush to receive the vaccine because they were good in health*,” and “*I did not see the importance of vaccination*”. One participant expressed a stronger negative view, saying:

*“Three people died in our house but I have never been vaccinated and yet I am alive, this leads me to say that Ebola virus disease did not exist*.”

- Non-vaccinated male in Mabalako FGD

Even perceptions of the vaccine among the vaccinated group were not always positive. For example, one participant pointed out:

*“The vaccine was not necessary…but anyways we were required to take it… [I] didn’t like it because our body is different from white people’s.”*

- Vaccinated male Community leader, Butembo FGD

#### Religious influence.

Faith-based perspectives, particularly beliefs in divine protection, were seen by many as alternatives to vaccination or EVD treatment. Several participants linked the Ebola vaccine to negative religious associations, such as satanism or demonic influence. For some, the act of getting vaccinated was perceived as a sign of not trusting in divine protection, with the belief that taking the vaccine could lead individuals down an “*evil*” path. As one participant explained:

*“People think that praying can cure certain diseases without using additional methods like vaccination. For others, their religion prohibits them [from getting vaccinated]"*

- Response Leader, Butembo KII

Conversely, some participants noted that in areas where the response worked closely with religious leaders, acceptance and uptake of the vaccine within religious groups significantly improved. These participants stressed the importance of understanding the community’s values and knowledge, and tailoring communication to align with their beliefs.

*“What I encourage in all the strategies that we have put in place is to continue with the awareness campaign with a particular focus on the leaders. We’ve reached out to people whose religions do not allow vaccination. But through their religious leaders, we were able to vaccinate a significant number of them."*

- Response Leader, Beni KII

### Convenience

Factors that make vaccination difficult, inaccessible, or costly influenced community perceptions and attitudes towards the Ebola vaccine and vaccination strategy.

#### Availability and cost of vaccine.

Concerns were raised regarding the availability of the Ebola vaccine, with limited supplies being a recurring issue. Some respondents noted that, despite the vaccine being advertised as free, they were asked to pay a fee to get it in certain instances. Additionally, there was a widespread belief that when supplies were scarce, the affluent or those with connections to response workers or political authorities were the ones who were able to access the vaccine. As one participant stated:

“*The second batch [of vaccine doses] was not accessible to everyone.* *It was the political authorities and the rich who benefited.”*

- Non-vaccinated male, Butembo FGD

#### Improvement of community sensitization efforts.

A common concern was the use of foreign or outsider workers who did not speak the local languages and which participants felt hindered effective community sensitization. To improve trust and communication, many participants suggested that sensitization efforts should be conducted in local languages and by local community members. One participant noted:

*“They didn’t speak any local languages apart from Lingala, French and Swahili which is not from here…But if you bring me someone who speaks my language there will be good communication and you will manage to get me to understand your message.”*

- Male Religious Leader, Beni mixed vaccination status FGD

Participants also recommended utilizing a variety of communication channels, including community groups, religious leaders, local community leaders, and radio broadcasts in local languages. Additionally, it was suggested that the dates, times and locations of vaccination sessions should be widely publicized to ensure greater awareness and access. Many felt that local HCWs, whether involved in the response or not, should serve as role models for the community, given their established trust within the community. This stemmed from reports of HCWs and other frontline personnel who had chosen not to be vaccinated and spoke out against the vaccine.

“*I did not have a lot of confidence because they [HCWs] sensitized the others to take the vaccine, but they would advise their children and members of their family to not get vaccinated or even investigated.*”

- Male Community Leader, Mabalako mixed vaccination status FGD

Finally, participants emphasized the importance of conducting sensitization efforts before the arrival of vaccination teams, believing this would maximize community awareness and acceptance of the vaccine.

#### Practical considerations related to the vaccination process.

Participants also highlighted logistical challenges that made vaccination difficult. Long wait times at vaccination sites and the significant distances some people had to travel were seen as deterrents. Additionally, the vaccination process involved an investigational protocol, which required individuals to complete informed consent forms and undergo active safety monitoring for adverse events. These procedures were considered by many to be lengthy and complex, further discouraging participation in the vaccination campaign.

*“There was a very long queue, and we spent entire days in the scorching sun before getting the vaccine… Before receiving the vaccine, you had to fill out several forms.”*

- Vaccinated female in Butembo FGD

## Discussion

A central finding from this study is that low vaccine confidence, driven by concerns about the vaccine itself, mistrust in the institutions or individuals delivering it, and skepticism about its deployment, significantly affected acceptance of the Ebola vaccine. This mistrust, particularly in the context of the pre-licensure vaccination campaign that occurred under investigational protocols, likely compounded concerns that the experimental vaccine may not be safe. Many respondents noted the lengthy consent forms and the extended follow-up for adverse events, which contributed to perceptions that the vaccine was not fully trusted or tested. This perception, coupled with a legacy of political instability and distrust in both domestic and international actors perceived as outsiders, significantly undermined vaccine confidence [7]. As the vaccine is now licensed, such barriers may be mitigated in future implementation efforts.

This study builds upon prior research into the sociocultural dynamics that drove vaccine acceptance during the 2018–2020 outbreak in Eastern DRC [7,22]. Study findings also reflect broader vaccine hesitancy patterns documented during other vaccination efforts in the DRC, including COVID-19, cholera, and measles responses [23–26]. Across these contexts, mistrust in government and external actors, circulating misinformation, and perceptions of inequitable access have repeatedly undermined vaccine confidence, suggesting that Ebola vaccine acceptance reflects deep-rooted structural and historical dynamics rather than disease-specific concerns alone. Given the increasing frequency of EVD and other outbreaks of international concern in the DRC, and the important role that vaccines play in their control, our findings on vaccine confidence and acceptance provide critical insights useful for policymakers and outbreak response teams into how communities navigate vaccination decisions in high-risk, conflict-affected settings.

While previous quantitative studies have reported overall high vaccine uptake among HCWs in DRC, our qualitative findings revealed more complex attitudes and motivations for vaccination [14,15]. Many reported being vaccinated due to the perceived necessity for protection in their line of work. For others, vaccine uptake was driven more by institutional mandates rather than by perceived benefits, suggesting a dynamic of coerced compliance rather than voluntary participation resulting from confidence in the vaccine’s effectiveness. Similar tensions between professional obligation, autonomy, and trust have been documented during COVID-19 vaccination campaigns in the DRC [27]. This interplay between confidence in the vaccine itself, confidence in the voluntary nature of the vaccination process and trust in the delivery system reinforces the need for more nuanced models of vaccine decision-making, particularly for HCWs who are both at high-risk and are well-positioned to influence vaccination intentions within their community [12]. Lessons from COVID-19 and influenza vaccination mandates indicate mixed HCW attitudes, with concerns about autonomy and trust in institutions affecting both uptake and public perception [28]. Future research is needed to explore the implications and ethical considerations surrounding mandatory Ebola vaccination policies for HCWs and other individuals at occupational risk and their influence on community vaccine acceptance.

Our results suggest that widespread misinformation about the vaccine’s potential to cause death, infertility, and long-term health effects, particularly for pregnant women, fueled hesitancy. These rumors were exacerbated by inconsistent messaging, insufficient and ineffective communication strategies. A key theme that emerged was the need for clear, transparent, and trusted information about the vaccine from reliable sources, including balanced information detailing both the advantages and potential risks of the vaccine. This underscores the critical need for transparent communication strategies that empower individuals to make informed decisions, supporting findings from similar studies [14,15]. As misinformation continues to play a significant role in shaping public perception of vaccines, the delivery of accurate, evidence-based information through trusted and competent local figures is essential.

Some community members expressed complacency toward vaccination, either because they believed they were not at risk or because they had survived previous outbreaks. Others cited religious beliefs as a protective factor against EVD, perceiving divine intervention as more trustworthy than vaccination [29]. Future research should explore the willingness to be vaccinated in contexts of perceived low disease risk, such as during post-outbreak preventive vaccination campaigns. For example, previous calls for the preventive vaccination of contacts of Ebola virus disease survivors to interrupt transmission in areas with recurrent outbreaks, highlight the need to examine the acceptability of such strategies [9].

Religious beliefs also emerged as a complex factor influencing vaccine acceptance. While some individuals saw faith-based protection as an alternative to vaccination, others recognized the potential of religious leaders to encourage vaccine uptake. Faith leaders and community elders were often seen as more trustworthy than official health authorities, a pattern observed across vaccine contexts, including cholera and COVID-19, in the DRC and elsewhere in sub-Saharan Africa [29,30]. While some religious messages and figures discouraged vaccination, others played a critical role in encouraging uptake. The dual role of religion highlights its importance in either amplifying or mitigating hesitancy. Notably, the quantitative phase of this study found that higher religiosity was associated with greater vaccine uptake, suggesting that religious engagement may support acceptance [15]. Future vaccination campaigns in similar settings where religion is deeply ingrained should therefore carefully consider the importance of aligning public health messages with local religious values to maximize success.

Logistical barriers, including long wait times, distant vaccination sites, and burdensome consent processes hindered access to vaccination. Some participants viewed the ring vaccination strategy, focused on the vaccination of social contacts of EVD cases, as discriminatory. While others believed that only the elite or politically connected received timely access or a more efficacious version of the vaccine. These barriers reinforced broader frustrations with the inequities perceived in the Ebola response that have been previously documented [31]. Ring vaccination was a pragmatic response to limited vaccine supply and an expanding outbreak, but its rationale was often unclear to communities, contributing to perceptions of favoritism. While population-based approaches such as geographic or mass vaccination may have been viewed as more equitable, they were not feasible under the circumstances. Strengthening communication around the rationale for vaccination strategies, and ensuring that these strategies are appropriately contextualized, is vital for fostering trust and improving uptake.

Vaccine perceptions were deeply entangled with political narratives. In a region scarred by decades of conflict, including violence reportedly perpetrated by armed groups from outside the country, participants frequently interpreted response and vaccination efforts through a lens of marginalization and control. Conspiracy theories about the true purpose of the vaccine were common, especially regarding international motives as others have noted elsewhere [7]. The disproportionate mobilization of resources for the Ebola outbreak control and vaccination efforts, compared to those allocated for other community needs including endemic diseases, along with the influx of international actors in the response, also contributed to alienation and suspicion.

Sustainable, community-driven vaccination efforts are essential for improving acceptance. Our findings support the growing recognition that continuous community engagement, particularly through the involvement of trusted and competent local leaders and influencers, builds long-term trust and reduces skepticism [32]. When vaccination campaigns are locally owned and embedded in the community’s social fabric, they are more likely to be effective and accepted. Localized health systems that reflect the cultural, social, and political realities of their communities are better equipped to foster adherence to health interventions [33]. In settings marked by institutional mistrust and a perception of foreign actors as outsiders, empowering local healthcare providers, religious leaders, and other trusted figures is key to ensuring health efforts resonate and gain legitimacy.

Especially in low-trust, conflict-affected settings, implementing health interventions through long-term, community-centered engagement can be challenging, yet remains essential. Our study reinforces that effective engagement must be sustained, adaptive, and integrated within broader health system strengthening efforts. Involving communities and FLWs, throughout both response and recovery phases creates feedback loops, improves communication, and promotes local ownership of public health strategies [32]. Future inquiry should also examine the relative impact of community engagement and social mobilization activities during outbreak response compared to preventive vaccination campaigns. Understanding these dynamics will help tailor engagement strategies to different phases of outbreak risk.

Findings from this study suggest several actionable strategies for improving vaccine uptake in conflict-affected outbreak contexts. These include co-developing culturally relevant communication materials with local communities; training and empowering trusted local leaders to serve as vaccine advocates; and ensuring equitable vaccine distribution to avoid perceptions of favoritism. Additionally, consent processes should be clearly explained and adapted to local contexts. Early in the outbreak, individually delivered consent under compassionate use protocols was perceived by some as burdensome or isolating. Later shifts to group education with individual consent were more acceptable, highlighting the importance of balancing ethical standards with culturally and logistically appropriate approaches. Finally, strengthening local health system capacity to reinforce long-term trust is crucial, as is sustaining community engagement beyond emergency phases to foster continuity and trust. Embedding anthropological and social science perspectives into response planning and evaluation may further support culturally grounded and responsive public health strategies.

## Limitations

While this study provides valuable insights into Ebola vaccine decision-making during the 2018–2020 outbreak in Eastern DRC, several limitations warrant consideration. The findings are context-specific and may not be generalizable to all EVD-affected settings, particularly given the unique sociopolitical dynamics in North Kivu. The qualitative design, while allowing for depth and nuance, involved a relatively localized sample, which may not fully represent the broader population or diverse subgroups.

Additionally, the retrospective nature of the study introduces potential recall bias, as participants were reflecting on experiences that may have evolved over time. The interviews were completed after the outbreak and during the early stages of the COVID-19 pandemic, which may have influenced how participants framed their experiences with Ebola vaccination. The heightened global attention to vaccination and public health during this period could have shaped perceptions of trust, risk, and institutional response, potentially prompting comparisons between the two outbreaks. Language barriers and researcher positionality may have influenced responses provided by participants, though these risks were mitigated through training local researchers, providing preferred language options for data collection, and culturally adapted protocols.

Despite these limitations, the study presents several important strengths. By capturing community perceptions during the pre-licensure phase of Ebola vaccine deployment, the research offers a critical baseline that future studies can build upon to assess how attitudes, trust, and hesitancy may evolve in post-licensure contexts or during preventive campaigns. This temporal positioning makes the study especially valuable for comparative analysis as the Ebola vaccine becomes more integrated into routine preparedness strategies [34].

Moreover, the qualitative approach enabled deep exploration of how trust, misinformation, and political context shape vaccine decisions; factors often missed in quantitative surveys. For example, while quantitative findings from the broader study showed high uptake and associations with trust, risk perception, and religiosity, our qualitative data revealed deeper complexities such as perceived coercion among HCWs and tensions between vaccine acceptance and religious beliefs. By capturing these lived experiences, this study adds important context to the survey data and highlights how sociopolitical dynamics shape vaccine decisions in fragile settings. Conducting the study in a conflict-affected setting added complexity but also provided a rare opportunity to examine vaccine acceptance under conditions of institutional fragility.

The active involvement of local researchers and community liaisons enhanced trust, cultural relevance, and data quality, offering a methodological model for research in similarly sensitive environments. Finally, by applying the WHO SAGE 3Cs model, the study offers a focused and policy-relevant analysis grounded in a framework that has been used in outbreak and emergency vaccination research [12]. Although more recent frameworks provide expanded constructs to capture additional dimensions of vaccine acceptance, the 3Cs remain foundational, and particularly suited for identifying primary behavioral determinants in high-risk, resource-constrained environments [35,36]. Our use of this model prioritized conceptual clarity and applicability, offering a strong foundation for future research to build upon.

## Conclusion

This study offers a critical, context-specific exploration of the sociocultural drivers of Ebola vaccine acceptance and hesitancy during the 2018–2020 outbreak in Eastern DRC. Conducted in a region marked by armed conflict, institutional fragility, and widespread mistrust, the study provides an in-depth understanding of how confidence, perceived risk, access, and socio-political dynamics influence vaccination decisions. Through FGDs and KIIs with diverse community members, including EVD survivors, healthcare workers, and religious leaders, the study captured a range of perspectives that reflect both individual experiences and broader systemic challenges.

Importantly, this research captured perceptions during the pre-licensure phase of Ebola vaccine deployment, offering a valuable baseline for future comparisons with licensed vaccine dose delivery and other vaccines deployed during the pre-licensure phase. As such, the findings not only illuminate key barriers and drivers at a critical historical moment but also lay the groundwork for understanding how perceptions may shift in response to changes in policy, delivery strategies, and levels of institutional trust.

Overall, the study reinforces the need for sustained and community-centered engagement strategies, transparent and balanced communication, integration of trusted local actors in planning and implementation for vaccine delivery, and the tailoring of public health messages to address deeply rooted concerns. Future vaccination campaigns, particularly in conflict-affected or low-trust settings, must go beyond biomedical considerations and actively engage with the social, political, and cultural contexts that shape community decision-making. In doing so, they will be better positioned to build lasting trust, counter misinformation, and support equitable access to life-saving vaccines.

## Supporting information

S1 File Inclusivity-in-global-research-questionnaire 1. (DOCX)
